# Approach to early grief: Report of two cases

**DOI:** 10.1192/j.eurpsy.2021.2074

**Published:** 2021-08-13

**Authors:** A. Sotillos Gómez, P. Marco Coscujuela, A. Hernández Mata

**Affiliations:** 1 Psychiatry, Hospital Universitario de Getafe, Getafe, Spain; 2 Psiquiatría, Hospital Universitario de Getafe, Getafe, Spain

**Keywords:** grief, mourning, illness

## Abstract

**Introduction:**

Early grief is a concept about which there is little literature. This generates difficulties in order to perform a differential diagnosis, as it poses complications to determine if the symptoms that the patient suffers are relative to the mourning or if they appear as part of a comorbid disorder.

**Objectives:**

To assess the difficulty in discriminating when accompaniment is necessary and when the patient can benefit from pharmacological, psychotherapeutic or combined treatment.

**Methods:**

Patients’ data is obtained from their medical history as well as psychological interviews carried out during the process.

**Results:**

32-year-old woman, with a previous history of depression. The patient was living abroad when her father was diagnosed with a terminal illness, so she decided to return home, making a radical change in her life. She is currently facing the functional deterioration of her father, who is rapidly getting worse. The patient shows symptoms of anxiety, tendency to cry and apathy. 34-year-old woman, with no history in Mental Health. As a result of her father’s illness, the patient develops a clinical manifestation of anxiety and low spirits. After one year, the clinic is maintained according to the variations in the health of her father. She also reports problems concentrating, fatigue, ruminative thoughts and structured autolithic ideas. Finally, she is referred to begin a psychotherapeutic follow-up.

**Conclusions:**

Bearing in mind that we are facing an increase in diagnoses of terminal illnesses, I consider it is necessary to reflect on this concept in order to provide a better response to patients.

**Disclosure:**

No significant relationships.

